# Two Decades of Progress in Cardiovascular Health: Declining Mortality and Years of Life Lost from Acute Myocardial Infarction in Poland

**DOI:** 10.3390/jcm15020838

**Published:** 2026-01-20

**Authors:** Monika Burzyńska, Piotr Jankowski, Małgorzata Pikala

**Affiliations:** 1 Department of Epidemiology and Biostatistics, Medical University of Lodz, 90-752 Lodz, Poland; malgorzata.pikala@umed.lodz.pl; 2 Department of Internal Medicine and Geriatric Cardiology, Centre of Postgraduate Medical Education, 01-813 Warszawa, Poland; piotrjankowski.eu@gmail.com; 3 Department of Epidemiology and Population Health, School of Public Health, Centre of Postgraduate Medical Education, 01-826 Warszawa, Poland

**Keywords:** acute myocardial infarction, cardiovascular mortality, years of life lost, standardised death rate, mortality trends

## Abstract

**Background**: The aim of the study was to assess trends in mortality and years of life lost due to acute myocardial infarction (AMI) in Poland between 2000 and 2023. **Methods**: We analysed aggregated, fully anonymised registry data comprising 431,793 death certificates of Polish residents who died from acute myocardial infarction (AMI). Standardised death rates (SDR) were calculated. Premature mortality was assessed using the Standard Expected Years of Life Lost (SEYLL). Trends were evaluated with joinpoint regression, estimating annual percentage change (APC) and average annual percentage change (AAPC). **Results**: During the study period, AMI deaths declined from 28,737 in 2000 to 11,328 in 2023, a 2.5-fold reduction. SDR decreased from 115.9 to 31.2 per 100,000, with an overall AAPC of −5.6% (*p* < 0.05). Declines were greater in men (AAPC −5.7%) than in women (−5.3%). SEYLL decreased from 608,488 years in 2000 to 191,476 years in 2023, representing a >70% reduction, with similar relative declines in men and women. SEYLLd fell from 21.2 to 16.9 years, indicating improved survival and older age at death. A temporary stagnation was observed between 2017 and 2020, followed by renewed steep declines thereafter. **Conclusions**: AMI mortality and YLL in Poland decreased markedly in the study period, reflecting advances in secondary prevention and public health.

## 1. Introduction

Acute myocardial infarction (AMI) is the most serious manifestation of coronary artery disease and continues to be a major public health concern. In most cases, it is caused by acute obstruction of coronary blood flow, most commonly due to rupture or erosion of an atherosclerotic plaque, followed by thrombus formation [1]. Despite substantial progress in prevention and treatment, reflected in the contemporary European Society of Cardiology (ESC) Guidelines on the management of acute coronary syndromes, AMI remains responsible for a considerable proportion of global cardiovascular mortality and disability [2,3]. Epidemiological evidence indicates that global prevalence of myocardial infarction increases markedly with age, reaching 9.5% in individuals over 60 years compared with 3.8% in those under 60 [4]. In Poland the prevalence of AMI is estimated at 1.3% of the population [5]. Classical risk factors—including hypertension, diabetes mellitus, dyslipidaemia, and smoking—remain the leading drivers of AMI incidence. More recently, obesity, metabolic syndrome, and sedentary lifestyle have contributed to the persistently high burden of ischaemic heart disease worldwide [6]. Importantly, AMI is associated not only with high short-term case fatality but also with premature mortality, making it a major determinant of years of life lost [7].

According to the Global Burden of Disease study, ischaemic heart disease—including AMI—accounted for almost 9 million deaths in 2021, representing the single most important cause of global mortality [8]. International data demonstrate that mortality from AMI has declined over the past decades, primarily owing to advances in acute reperfusion therapy, pharmacological treatment, and improved secondary prevention [9,10]. However, the pace of these improvements has been uneven. While Western Europe and North America have experienced consistent reductions, Central and Eastern Europe—including Poland—have historically carried higher rates of cardiovascular mortality [11,12]. In approximately one in ten patients, myocardial infarction is fatal during the acute phase, and nearly one in five patients die within the first year following the event. It is estimated that more than 80,000 myocardial infarctions occur annually in Poland. Men are more frequently affected than women (62.5% vs. 37.5%) [5]. Despite the considerable progress in interventional cardiology and the favourable outcomes of myocardial infarction treatment, the incidence of this condition in Poland remains 30–50% higher than in Western European countries and the European Union overall [13]. In 2022, Poland’s age-standardised death rate due to acute myocardial infarction (33.9 per 100,000) was slightly below the European Union average (36.0 per 100,000), placing the country in the mid-range among EU member states. To address these challenges, the National Health Fund introduced the managed care for acute myocardial infarction survivors—MACAMIS (KOS-Zawał) programme in 2017. This nationwide initiative was designed to provide comprehensive, structured post-AMI care, including acute treatment, complex revascularisation, cardiac rehabilitation, scheduled follow-up visits, and prevention of sudden cardiac death [14]. Observational analyses have demonstrated that participation in MACAMIS is associated with substantial survival benefits, with a 30% reduction in one-year mortality (HR 0.70, 95% CI 0.62–0.80), and the effect persists beyond the programme duration. Importantly, cardiac rehabilitation (HR 0.34) and structured outpatient care (HR 0.42) were identified as the most influential components contributing to mortality reduction [15].

In Poland, ischaemic heart disease still remains the leading cause of death, although recent years have brought significant improvements in outcomes [16,17]. Monitoring long-term mortality trends and years of life lost due to AMI is essential to understand the effectiveness of public health interventions, evaluate health system performance, and identify populations at greatest risk.

The aim of this study was to examine long-term trends in AMI mortality in Poland between 2000 and 2023, with particular focus on standardised death rates and years of life lost.

## 2. Materials and Methods

The study material consisted of aggregated, fully anonymised registry data comprising 431,793 death certificates of all Polish inhabitants who died due to acute myocardial infarction between 2000 and 2023, classified according to the International Statistical Classification of Diseases and Health Related Problems, Tenth Revision (ICD-10 code I21). The data were provided by Statistics Poland. The procedure of coding causes of death in Poland is performed in a similar way to the one carried out in the majority of countries in the world, by basing it on the so-called primary cause of death, or the disease which triggered a pathological process, leading to death.

The authors calculated standardised death rates (SDR). The standardisation procedure was performed with the use of the direct method, in compliance with the European Standard Population, updated in 2012 [18].

Years of life lost were calculated and analysed by the method described by Christopher Murray and Alan Lopez in Global Burden of Disease (GBD) [19]. The Standard Expected Years of Life Lost (SEYLL) index is used to calculate the number of years of life lost by the studied population in comparison to the number of years lost by the referential (standard) population.

There are some methods of calculating lost years of life, and the main difference between them is a point of reference, i.e., the level of mortality which is considered “ideal”. In the GBD 2010 study, World Health Organization (WHO) experts recommend using life tables based on the lowest noted death rate for each age group in countries with populations above 5 million [20].

In this study, the SEYLL index was calculated according to the following formula:

$$SEYLL=\sum_{x=0}^{l}d_{x}e_{x}^{*}$$

where $e_{x}^{*}$—life expectancy, based on GBD 2010 life tables, d_x_—number of deaths at age x, x—age at which the person died, l—last age which the population reaches.

The authors also applied the SEYLL per person (SEYLL_p_) index, which is a ratio of SEYLL and the size of population, calculated per 100,000 inhabitants, and the SEYLL per death (SEYLL_d_) index, being a ratio of SEYLL and the number of deaths due to a particular cause, i.e., it expresses the number of YLL calculated per one dead person [21].

The analysis of time trends has been carried out with joinpoint models and Joinpoint Regression Program (version 4.8.0.1), a statistical software package developed by the U.S. National Cancer Institute for the Surveillance, Epidemiology and End Results Program [22].

Joinpoint regression model is an advanced version of linear regression y = bx + a, where b is the slope coefficient, a is the y-intercept, y is a measure evaluated in the study and x is a calendar year. Time trends were determined with the use of segments joining in joinpoints where trend values significantly changed (*p* < 0.05). In order to determine whether the changes were statistically significant, the Monte Carlo Permutation method was applied.

In addition, the authors also calculated Annual Percentage Change (APC) for each segment of broken lines and Average Annual Percentage Change (AAPC) for the whole study period with corresponding 95% confidence intervals (CI).

Annual Percent Change is used to characterise trends in death rates over time and it was calculated according to the following formula:

$$APC=100*({exp}^{b}-1)$$

where b—the slope coefficient.

Average Annual Percent Change (AAPC) is a summary measure of the trend over a pre-specified fixed interval. It allows us to use a single number to describe the average APCs over a period of multiple years. It is valid even if the joinpoint model shows that there were changes in trends during those years. It is computed as a weighted average of the APCs from the joinpoint model, with the weights equal to the length of the APC interval [23].

$$AAPC=\left\{exp\left(\frac{\sum w_{i}b_{i}}{\sum w_{i}}\right)-1\right\}\times 100$$

where b_i_—the slope coefficient for each segment in the desired range of years, w_i_—the length of each segment in the range of years.

Statistical analyses were performed using the Statistica (data analysis software system), version 13 (TIBCO Software Inc., San Ramon, CA, USA). 

## 3. Results

Between 2000 and 2023, a total of 431,793 individuals died in Poland due to acute myocardial infarction, including 266,765 men (61.8%) and 165,028 women (38.2%). The number of deaths decreased 2.5-fold, from 28,737 in 2000 to 11,328 in 2023 (Table 1). A greater decline was observed among men (2.7-fold, from 18,299 in 2000 to 6756 in 2023) than among women (2.3-fold, from 10,438 to 4572 deaths, respectively).

Expressed per 100,000 population, the standardised death rate (SDR) fell from 115.9 in 2000 to 31.2 in 2023. The average annual percentage change (AAPC) in the years 2000–2023 was −5.6% (*p* < 0.05) (Table 2).

**Figure 1:**
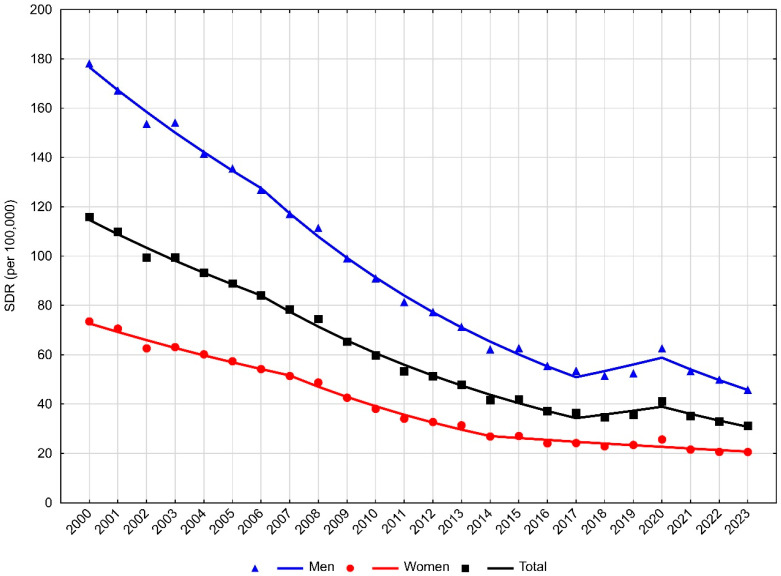
Trends in standardised death rates due to acute myocardial infarction in Poland, 2000–2023.

This downward trend slowed between 2017 and 2020, during which changes were not statistically significant. After 2020, SDRs began to decline again, with an annual percentage change (APC) of −7.5% (*p* < 0.05) (Table 3, Figure 1).

Among men, the SDR decreased from 178.2 in 2000 to 45.8 in 2023 (Table 1). The rate of decline in 2000–2006 was −5.3% per year, accelerating to −8.0% per year between 2006 and 2017 (*p* < 0.05). From 2017 to 2020, changes were not statistically significant. After 2020, the SDR among men resumed its decline at −10.7% annually (*p* < 0.05) (Table 3, Figure 1). For the entire period 2000–2023, the AAPC in men was −5.7% (*p* < 0.05).

Among women, the SDR declined from 73.5 in 2000 to 20.6 in 2023 (Table 1). The AAPC was slightly lower than in men, at −5.3% (*p* < 0.05). A statistically significant decrease was observed across the whole study period. The pace of decline was −4.8% per year in 2000–2007, increasing to −8.8% in 2007–2014, then slowing to −2.9% after 2014 (Table 3, Figure 1).

**Figure 2:**
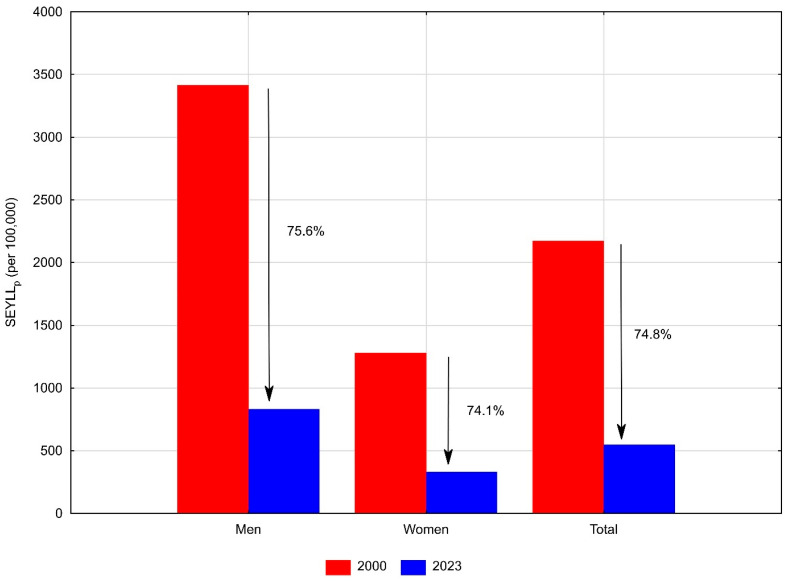
Standard expected years of life lost (SEYLL_p_) due to acute myocardial infarction in Poland in 2000 and 2023.

Premature mortality due to acute myocardial infarction directly contributes to the burden of years of life lost. The number of standard expected years of life lost (SEYLL) was 608,488 in 2000, and decreased to 191,476 in 2023 (Table 2). Approximately 70% of total years of life lost were attributable to male deaths, and 30% to female deaths. Among men, SEYLL decreased from 432,864 in 2000 to 132,208 in 2023. Per 100,000 male population, SEYLLp dropped by 75.6%, from 3414.2 in 2000 to 832.5 in 2023 (Table 2, Figure 2).

The annual percentage change over the entire study period was −5.9% (*p* < 0.05) (Table 3). As with the SDR, the downward trend in SEYLLp slowed between 2017 and 2020, during which changes were statistically insignificant. After 2020, SEYLLp resumed its decline at a rate of −8.7% per year (*p* < 0.05).

Among women, SEYLL decreased from 175,625 in 2000 to 59,269 in 2023. Between 2000 and 2023, SEYLLp declined by 74.1%, from 1280.6 to 331.5 per 100,000 women (Table 2, Figure 2). The annual percentage change for the entire period was −5.7% (*p* < 0.05) (Table 3). Similarly to men, after declines in SEYLLp between 2000–2007 (APC = −5.5%, *p* < 0.05) and 2007–2016 (APC = −8.4%, *p* < 0.05), a period of statistically insignificant change occurred from 2016 to 2020. Following 2020, SEYLLp began to decline again at −6.6% annually (*p* < 0.05).

The study also calculated SEYLLd (per death) coefficients. In 2000, each person who died due to acute myocardial infarction lost on average 21.2 years of life, while in 2023 the figure was 16.9 years (AAPC = −0.9%) (Table 2 and Table 3). A higher number of years lost per death was observed among men. In this group, SEYLLd decreased from 23.7 in 2000 to 19.6 in 2023. Statistically significant changes occurred during the periods 2000–2015 (APC = −0.8%) and 2021–2023 (APC = −4.4%). The average annual percentage change for the entire period was not statistically significant.

Among women, the average number of years of life lost per death was 16.8 years in 2000, and 13.0 years in 2023 (AAPC = −0.9%, *p* < 0.05).

## 4. Discussion

The present study analyses nationwide mortality data in Poland from 2000 to 2023, focusing on standardised death rates and years of life lost. By quantifying long-term trends and sex differences, it provides updated evidence on the evolving burden of AMI in Poland and contributes to a broader understanding of cardiovascular epidemiology in Central and Eastern Europe. Our findings demonstrate a marked decline in AMI mortality over the study period. The number of deaths fell by more than half, while age-standardised death rates decreased nearly threefold. These improvements were evident in both men and women, although the relative reduction was greater in men. The declines translated into substantial reductions in years of life lost, both per person (SEYLLp) and per death (SEYLLd), underscoring progress not only in overall survival but also in premature mortality. The temporary stagnation observed between 2017 and 2020, including a non-significant increase in SDR, SEYLLp and SEYLLd, likely reflects a short-term flattening of long-term declining trends rather than a reversal. Although an increase in AMI mortality in 2020 has been reported in some studies and is commonly attributed to the COVID-19 pandemic, resulting from the sudden overload of the healthcare system, the broader plateau observed in our analysis precedes the pandemic and therefore cannot be explained by it alone. These indicators are particularly sensitive to shifts in age at death and population ageing, and their fluctuations may be driven by demographic dynamics and cumulative lifetime exposure to cardiovascular risk factors rather than abrupt changes in AMI incidence or case fatality. It is worth noting that in-hospital mortality decreased dynamically at the beginning of the century (e.g., from 9.1% in 2009 to 8.0% in 2012) but in 2018, it amounted to 8.4% [5]. It seems that the impact of implementing invasive treatment of heart attacks has disappeared, which contributed to the change in the trend.

The decline in AMI mortality in Poland is consistent with trends observed across Europe and other high-income countries. Since the late 20th century, Western European nations have reported sustained decreases in cardiovascular mortality, driven by advances in acute care, secondary prevention, and improved risk factor management [10,24]. In Central and Eastern Europe, however, progress began later and initially at a slower pace, reflecting historical differences in health systems and baseline cardiovascular risk profiles [11,12]. A Lithuanian population-based study, for example, reported significant reductions in AMI morbidity in adults aged 25–64 years, with mortality from ischaemic heart disease (IHD) falling markedly in men but less so in women [25]. Similarly, a recent German analysis (1998–2023) showed a consistent decline in coronary heart disease (CHD) mortality, although the pace of improvement slowed in the past decade, particularly in those aged 60–74 years. Importantly, a greater reduction was observed in AMI than in chronic forms of CHD, highlighting the role of advances in acute care [26]. Norwegian data likewise confirm long-term progress, with AMI mortality in 2021 lower than in the 1950s, reflecting improvements in both incidence and case fatality [27]. Against this background, Poland has achieved particularly steep gains since the early 2000s, coinciding with the establishment of a nationwide percutaneous coronary intervention (PCI) network, broader uptake of evidence-based pharmacotherapy, and preventive initiatives [28,29,30]. The first PCI in Poland was successfully performed in 1985 at the Institute of Cardiology in Warsaw-Anin, only eight years after Andreas Grüntzig introduced the technique in Zurich. During the 1990s, PCI procedures were gradually implemented in selected tertiary centres, but their availability remained limited, and thrombolysis continued to dominate as the standard reperfusion strategy. A major breakthrough occurred in the early 2000s with the establishment of a nationwide network of 24/7 catheterisation laboratories dedicated to primary PCI in ST-segment elevation myocardial infarction (STEMI). This so-called “Polish STEMI network” became fully operational by 2003 and was among the first comprehensive systems of its kind in Europe [31,32]. Since then, the volume of PCI in Poland has increased dramatically, with primary PCI adopted as the predominant reperfusion strategy. By the mid-2000s, more than 80% of STEMI patients were treated with primary PCI, a proportion that exceeded many Western European countries at that time [32]. This achievement contributed substantially to the pronounced decline in acute myocardial infarction (AMI) mortality observed in Poland during the first two decades of the 21st century. Beyond advances in interventional cardiology, successive updates to the universal definition of myocardial infarction—most notably those introduced in 2012 and 2018 [33]—also warrant consideration when interpreting long-term mortality trends. These revisions expanded diagnostic criteria, particularly through the increased use of high-sensitivity cardiac troponins, leading to the reclassification of some patients who would previously have been diagnosed with unstable angina. As these patients generally present with less severe clinical courses and more favourable prognoses, such changes may have influenced observed case fatality rates. However, it is important to note that revisions to the universal definition of myocardial infarction are more likely to increase diagnostic sensitivity and case ascertainment rather than to directly reduce mortality. Consequently, the sustained and substantial decline in AMI mortality observed in Poland is more plausibly attributable to improvements in acute revascularisation strategies, including the widespread implementation of primary percutaneous coronary intervention, alongside broader access to evidence-based pharmacotherapy and advances in secondary prevention. Together, these developments have translated into durable reductions in AMI mortality over the past two decades.

**Figure 3:**
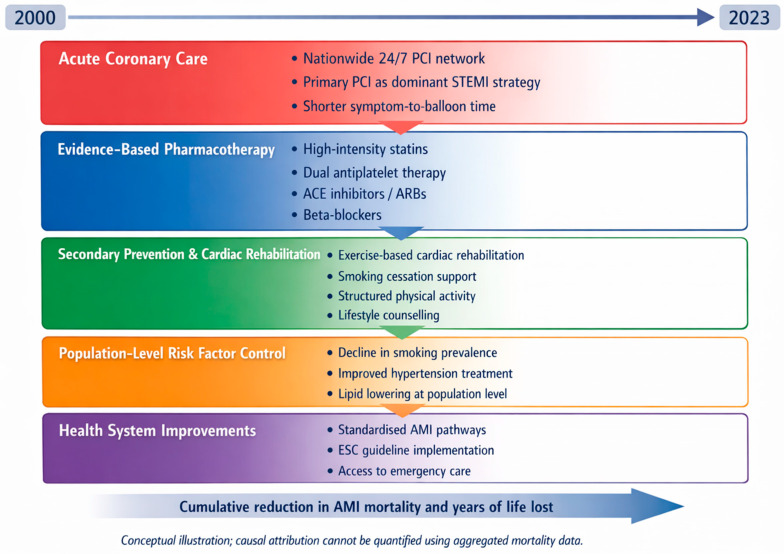
Factors contributing to the decline in mortality due to acute myocardial infarction in Poland in 2000 and 2023.

Beyond advances in acute reperfusion therapy, sustained reductions in AMI mortality critically depend on the effectiveness of secondary prevention. Contemporary strategies encompass evidence-based pharmacological treatment, including dual antiplatelet therapy, high-intensity statins (atorvastatin, rosuvastatin), β-blockers, and renin–angiotensin system inhibitors (ACE inhibitors or angiotensin receptor blockers), all of which are routinely used in post-AMI care in Poland in accordance with ESC guidelines for acute coronary syndromes and cardiovascular prevention [34,35]. Equally important are non-pharmacological interventions, particularly lifestyle modification, regular physical activity, and structured cardiac rehabilitation. Exercise-based cardiac rehabilitation has been consistently shown to reduce recurrent cardiovascular events, improve functional capacity, enhance adherence to medical therapy, and lower long-term mortality after myocardial infarction [36]. Cardiac rehabilitation has been shown to reduce recurrent cardiovascular events, improve functional capacity, enhance adherence to medical therapy, and lower long-term mortality after myocardial infarction. Despite its proven benefits, participation in cardiac rehabilitation remains suboptimal in many countries, underscoring the need for broader implementation and improved access to structured secondary prevention programmes. Recent evidence highlights that integrating pharmacological treatment with supervised exercise training and long-term lifestyle support yields the greatest prognostic benefit in patients with coronary artery disease and post-AMI status [37]. To address the multifactorial nature of the observed decline in AMI mortality, Figure 3 summarises key secondary prevention and healthcare system interventions implemented in Poland over the past two decades.

While causal attribution is not possible in this registry-based analysis, prior modelling and epidemiological studies consistently demonstrate that improvements in acute coronary care, evidence-based pharmacotherapy, cardiac rehabilitation, and risk factor control act synergistically to reduce post-AMI mortality [38].

Despite overall improvements, sex disparities persist. Men accounted for nearly two-thirds of AMI deaths and approximately 70% of years of life lost. SDRs were consistently higher among men, confirming their greater overall population burden. These disparities may reflect differences in lifetime exposure to risk factors, symptom presentation, access to invasive treatment, participation in secondary prevention and cardiac rehabilitation programmes and fundamental epidemiological differences between men and women rather than inequalities in access to acute care alone. Importantly, this study evaluates mortality, not incidence or case fatality. Population-level sex disparities in AMI mortality observed in our study are consistent with well-documented differences in age at disease onset and hospitalisation patterns. Nationwide registry data from Poland indicate that in 2018 men accounted for nearly two-thirds of all hospitalisations for acute myocardial infarction and were, on average, almost 7 years younger at admission than women (mean age 66.3 vs. 73.1 years). Although admission rates were substantially higher among men (255.2 vs. 142.5 per 100,000 inhabitants), women were hospitalised at significantly older ages, when the risk of death from cardiovascular causes is inherently higher due to multimorbidity, frailty, and reduced physiological reserve [5].

Consequently, higher AMI mortality rates among men at the population level primarily reflect their greater incidence and earlier onset of disease, whereas deaths among women occur later in life and contribute disproportionately to mortality in older age groups. This age-structured pattern helps explain the persistence of sex differences in AMI mortality despite improvements in acute care and secondary prevention. This is consistent with prior evidence from Poland and other Central and Eastern European countries [11].

The reductions in SEYLL and SEYLLp observed in this study emphasise progress in reducing premature mortality, with >70% declines in both sexes. This complements crude mortality statistics by highlighting the societal burden of AMI in terms of productive life years lost. Earlier Polish studies had identified ischaemic heart disease as a major contributor to years of life lost, especially among working-age men [13,39]. Our findings extend this trajectory, demonstrating sustained improvements into the third decade of the 21st century. The decrease in SEYLLd—from 21.2 to 16.9 years per death—indicates that individuals now dying from AMI are older, reflecting improved survival among younger patients.

A notable feature of our results is the stagnation of mortality decline between 2017 and 2020, followed by a steep resumption thereafter. This pattern may reflect several factors. First, the benefits of earlier advances, such as the PCI network and widespread statin therapy, may have plateaued. Second, the rising prevalence of obesity, diabetes, and other metabolic disorders in Poland may have offset some of the gains [40]. Germany has reported a similar slowing of progress in recent years [26], and comparable plateaus have been noted in the United States and other European nations, often linked to persistent risk factors and systemic pressures [8,41]. The sharp decline after 2020 suggests resilience within the Polish healthcare system but also underscores the importance of preparedness for future disruptions. Future research should focus on identifying population subgroups that benefit least from current prevention strategies, improving uptake of cardiac rehabilitation, and integrating lifetime risk assessment into routine care. Linking registry data with clinical and socioeconomic datasets may further enhance understanding of long-term AMI outcomes.

### Limitations

This study has several limitations. First, it is based on death certificate data, which may be subject to misclassification and temporal changes in cause-of-death coding. Second, the use of aggregated registry data and the lack of individual-level clinical information precluded adjustment for treatment patterns, comorbidities, cardiovascular risk factors, and socio-economic status. Third, evolving diagnostic criteria for myocardial infarction over time may have influenced mortality attribution. Nevertheless, the use of nationwide, population-based mortality data combined with joinpoint regression provides a robust framework for assessing long-term trends in AMI mortality.

## 5. Conclusions

Our nationwide analysis of acute myocardial infarction mortality in Poland from 2000 to 2023 reveals major progress in cardiovascular health, with more than a twofold reduction in absolute deaths, a nearly threefold decline in standardised mortality rates, and a substantial decrease in years of life lost. These gains reflect improvements in acute cardiac care and secondary prevention, yet persistent sex disparities and a temporary stagnation in mortality decline highlight remaining challenges. Sustaining this positive trend will require continued investment in population-level prevention, effective control of cardiovascular risk factors, and equitable access to high-quality acute and long-term care. Ongoing surveillance of mortality and years of life lost should guide national policies to further reduce the burden of cardiovascular disease in Poland.

## Figures and Tables

**Table 1:** Number of deaths and standardised death rates (SDR) due to acute myocardial infarction in Poland, 2000–2023.

Year	Total		Men		Women	
	Number of Deaths	SDR (per 100,000)	Number of Deaths	SDR (per 100,000)	Number of Deaths	SDR (per 100,000)
2000	28,737	115.9	18,299	178.2	10,438	73.5
2001	27,675	109.9	17,447	167.2	10,228	70.6
2002	25,417	99.4	16,168	153.6	9249	62.6
2003	25,476	99.5	16,083	154.2	9393	63.1
2004	24,371	93.2	15,224	141.6	9147	60.2
2005	23,547	88.9	14,637	135.5	8910	57.4
2006	22,930	84.1	14,261	127.0	8669	54.2
2007	21,636	78.3	13,248	117.1	8388	51.4
2008	21,021	74.5	12,829	111.4	8192	48.8
2009	18,666	65.3	11,334	99.0	7332	42.6
2010	17,637	59.8	10,900	91.0	6737	38.1
2011	16,112	53.4	9902	81.4	6210	34.1
2012	15,944	51.3	9811	77.3	6133	32.8
2013	15,109	47.9	9125	71.3	5984	31.4
2014	13,469	41.6	8182	62.2	5287	26.9
2015	13,813	41.9	8369	62.7	5444	27.1
2016	12,477	37.2	7535	55.5	4942	24.1
2017	12,399	36.4	7331	53.6	5068	24.2
2018	12009	34.7	7135	51.5	4874	22.9
2019	12,606	35.7	7552	52.5	5054	23.5
2020	14,735	41.2	9181	62.6	5554	25.7
2021	12,767	35.2	8046	53.4	4721	21.6
2022	11,926	33.0	7413	49.9	4513	20.7
2023	11,328	31.2	6756	45.8	4572	20.6
Total	431,793		266,765		165,028	

**Table 2:** Time trends of SDR, SEYLLp and SEYLLd due to acute myocardial infarction in Poland, 2000–2023—joinpoint regression analysis.

	Number of Joinpoints	Years	APC (95% CI)	AAPC (95% CI)
Total				
SDR	3	2000–2006	−5.1 * (−6.4; −3.7)	−5.6 * (−6.7; −4.4)
		2006–2017	−7.8 * (−8.4; −7.2)	
		2017–2020	4.3 (−4.2; 13,6)	
		2020–2023	−7.5 * (−11.4; −3.5)	
SEYLL_p_	3	2000–2006	−5.5 * (−6.8; −4.1)	−5.8 * (−6.9; −4.7)
		2006–2017	−8.0 * (−8.6; −7.4)	
		2017–2020	5.2 (−3.1; 14.2)	
		2020–2023	−8.3 * (−12.0; −4.4)	
SEYLL_d_	3	2000–2003	−1.8 * (−3.6; −0.1)	−0.9 * (−1.5; −0.4)
		2003–2018	−1.0 * (−1.2; −0.8)	
		2018–2021	3.1 (−0.5; 6.8)	
		2021–2023	−4.9 * (−8.2; −1.5)	
Men				
SDR	3	2000–2006	−5.3 * (−6.7; −3.9)	−5.7 * (−6.7; −4.7)
		2006–2017	−8.0 * (−8.7; −7.4)	
		2017–2020	5.0 (−2.8; 13.3)	
		2020–2023	−10.7 * (−14.5; −6.7)	
SEYLL_p_	3	2000–2006	−5.6 * (−6.8; −4.4)	−5.9 * (−6.9; −4.9)
		2006–2017	−8.2 * (−8.7; −7.7)	
		2017–2020	5.7 (−1.7; 13.6)	
		2020–2023	−8.7 * (−12.0; −5.3)	
SEYLL_d_	3	2000–2015	−0.8 * (−0.9; −0.6)	−0.7 (−1.4; −0.1)
		2015–2018	−1.7 (−5.1; 1.8)	
		2018–2021	2.9 (−0.6; 6.5)	
		2021–2023	−4.4 * (−7.7; −1.1)	
Women				
SDR	2	2000–2007	−4.8 * (−6.5; −3.0)	
		2007–2014	−8.8 * (−11.0; −6.7)	−5.3 * (−6.0; −4.1)
		2014–2023	−2.9 * (−4.1; −1.6)	
SEYLL_p_	3	2000–2007	−5.5 * (−6.8; −4.1)	−5.7 * (−6.8; −4.6)
		2007–2016	−8.4 * (−9.5; −7.4)	
		2016–2020	0.9 (−4.3; 6.3)	
		2020–2023	−6.6 * (−11.4; −1.6)	
SEYLL_d_	2	2000–2017	−1.5 * (−1.6; −1.3)	−0.9 * (−1.5; −0.3)
		2017–2021	2.7 * (0.1; 5.4)	
		2021–2023	−3.7 (−8.6; 1.4)	

* statistically significant.

**Table 3:** Standard expected years of life lost due to acute myocardial infarction in Poland, 2000–2023.

Year	Total			Men			Women		
	SEYLL	SEYLL_p_ (per 100,000)	SEYLL_d_ (per Death)	SEYLL	SEYLL_p_ (per 100,000)	SEYLL_d_ (per Death)	SEYLL	SEYLL_p_ (per 100,000)	SEYLL_d_ (per Death)
2000	608,488	2171.7	21.2	432,864	3414.2	23.7	175,625	1280.6	16.8
2001	576,508	2053.5	20.8	408,729	3201.5	23.4	167,779	1224.6	16.4
2002	519,770	1851.0	20.4	371,448	2929.6	23.0	148,322	1081.2	16.0
2003	509,306	1825.4	20.0	361,810	2891.7	22.5	147,495	1075.0	15.7
2004	486,513	1707.1	20.0	343,788	2662.9	22.6	142,725	1020.8	15.6
2005	458,802	1617.4	19.5	324,272	2535.3	22.2	134,530	966.7	15.1
2006	449,289	1540.8	19.6	319,881	2394.0	22.4	129,408	917.5	14.9
2007	415,453	1425.0	19.2	293,155	2198.1	22.1	122,297	863.2	14.6
2008	397,478	1350.3	18.9	281,218	2078.4	21.9	116,261	817.3	14.2
2009	344,585	1175.9	18.5	240,921	1828.1	21.3	103,664	713.3	14.1
2010	329,453	1081.0	18.7	236,183	1693.9	21.7	93,270	632.5	13.8
2011	296,902	963.4	18.4	211,701	1506.0	21.4	85,201	567.5	13.7
2012	297,827	932.8	18.7	213,147	1447.9	21.7	84,680	547.9	13.8
2013	275,433	862.6	18.2	194,587	1322.3	21.3	80,845	518.5	13.5
2014	244,996	750.5	18.2	172,537	1156.2	21.1	72,459	447.4	13.7
2015	246,906	749.9	17.9	174,289	1155.1	20.8	72,616	445.6	13.3
2016	220,584	664.9	17.7	154,740	1027.5	20.5	65,844	395.2	13.3
2017	211,533	642.3	17.1	146,276	976.3	20.0	65,257	393.8	12.9
2018	205,565	613.9	17.1	142,012	937.0	19.9	63,553	374.3	13.0
2019	221,795	638.5	17.6	153,356	967.0	20.3	68,440	388.2	13.5
2020	268,463	748.0	18.2	191,769	1166.5	20.9	76,694	427.7	13.8
2021	239,435	647.5	18.8	173,034	1013.4	21.5	66,401	360.9	14.1
2022	216,807	597.4	18.2	153,158	920.7	20.7	63,649	347.5	14.1
2023	191,476	547.5	16.9	132,208	832.5	19.6	59,269	331.5	13.0

## Data Availability

The data presented in this study are available on request from the corresponding author.

## Abbreviations

The following abbreviations are used in this manuscript:

AAPC	Average Annual Percentage Change
AMI	Acute Myocardial Infarction
APC	Annual Percentage Change
CHD	Coronary Heart Disease
CI	Confidence Intervals
GBD	Global Burden Of Disease
ICD-10	International Classification Of Diseases, Tenth Revision
PCI	Percutaneous Coronary Intervention
SDR	Standardised death rates (SDR)
SEYLL	Standard Expected Years of Life Lost
SEYLL_d_	Standard Expected Years of Life Lost per death
SEYLL_p_	Standard Expected Years of Life Lost per population
STEMI	Segment Elevation Myocardial Infarction
WHO	World Health Organization
